# Platelet-to-white blood cell ratio as a predictor of postoperative outcomes in acute type A aortic dissection: a single-center retrospective analysis

**DOI:** 10.3389/fcvm.2025.1669151

**Published:** 2026-01-06

**Authors:** Biwen Yang, Yucheng Hou, Mingzhu Xu, Tingbo Jiang

**Affiliations:** 1 Department of Cardiovascular Surgery, The First Affiliated Hospital of Soochow University, Suzhou, Jiangsu, China; 2 Department of Cardiology, The First Affiliated Hospital of Soochow University, Suzhou, Jiangsu, China

**Keywords:** acute type A aortic dissection, platelet-to-white blood cell ratio, postoperative adverse events, short- and mid-term mortality, prognostic indicator

## Abstract

**Objective:**

To evaluate the predictive value of preoperative platelet-to-white blood cell ratio (PWR) for postoperative outcomes in patients with acute type A aortic dissection (ATAAD).

**Methods:**

In this single-center retrospective cohort study, 363 ATAAD patients undergoing emergency type II hybrid aortic arch repair between January 2021 and February 2024 were stratified by median PWR into low PWR (<13.259) and high PWR (≥13.259) groups. Clinical variables, operative details, and outcomes were collected. Primary outcome was in-hospital postoperative adverse events (PAEs) incidence; secondary outcomes included short- and mid-term mortality. Associations were analyzed using multivariable logistic regression and Cox proportional hazards models.

**Results:**

A considerably higher incidence of PAEs was observed in the low PWR (<13.259) relative to the high PWR (41.99% vs. 21.43%, *p* < 0.001) groups. Moreover, patients with low PWR showed increased in-hospital (13.81% vs. 3.30%, *p* = 0.001), 90-day (14.36% vs. 4.40%, *p* < 0.001), and 1-year (16.38% vs. 5.56%, *p* = 0.001) mortalities. Multivariable logistic regression detected low preoperative PWR as a distinct marker of PAEs. The areas under the curve for PWR in estimating PAEs was 0.705 (95% CI, 0.649–0.760). Cox regression analysis confirmed a significant association between low PWR and elevated short- and mid-term mortality.

**Conclusion:**

Preoperative low PWR independently predicts postoperative complications and mortality in ATAAD patients, serving as an accessible and cost-effective biomarker for risk stratification and clinical decision-making.

## 1 Introduction

Acute type A aortic dissection (ATAAD) is a low-incidence (2.1–16.3 cases/100,000 individuals) but high-mortality cardiovascular emergency (1). Previous studies have revealed that, without timely intervention, ATAAD is associated with a markedly elevated risk of mortality, increasing by approximately 1%–2% per hour within the first 48 h of symptom onset, culminating in a cumulative mortality rate up to 37% (2, 3). However, recent progress in diagnostic tools and therapeutic strategies has contributed to a substantial reduction in mortality, the International Registry of Acute Aortic Dissection (IRAD) found that the overall 48-hour mortality rate of ATAAD patients was 5.8%, with rates of 23.7% in medically managed patients vs. 4.4% in those receiving or planned for surgery (4). Therefore, immediate surgery is the treatment of choice for ATAAD. Nevertheless, postoperative mortality remains significant (11%–25%) (5, 6), and the incidence of postoperative adverse events (PAEs), such as postoperative infection, acute kidney injury (AKI), neurological disorders, respiratory complications, and cardiovascular diseases (CVD), remains high (7–9). These complications not only prolong patients’ hospital stay and increase medical costs but also significantly reduce their short and long-term survival.

Many studies indicate that coagulation dysfunction and inflammatory responses play crucial roles in aortic vascular injury and collectively contribute to ATAAD development (10, 11). Multiple clinical studies reveal that systemic coagulation and inflammation markers, white blood cell (WBC), C-reactive protein (CRP), platelet (PLT), and D-dimers are closely related to adverse prognoses in ATAAD patients (12–15). The neutrophil-to-lymphocyte ratio, systemic Immune-Inflammation Index, lymphocyte-to-monocyte ratio, and pan-immune-inflammation value are markers reflecting systemic inflammation, which have been independently correlated with clinical outcomes in ATAAD patients (11, 16, 17).

Recently, platelet-to-white blood cell ratio (PWR), a marker of systemic inflammatory response, has gained increasing recognition since its initial introduction by Toutouzas et al. It is derived by dividing the PLT by the WBC count and serves primarily as an index to quantify the inflammatory status of the host (18). Further studies have revealed its prognostic application in various pathological conditions, including prevalent chronic disorders (19, 20) and acute, life-threatening states (21, 22). Previous evidence has also linked preoperative PWR levels to 30-day mortality outcomes in patients undergoing surgery for ATAAD (23, 24). However, the relationship between PWR and the incidence of postoperative complications, as well as short- and intermediate-term mortality after ATAAD surgery, has not been fully elucidated.

Accordingly, this study investigates the association between preoperative PWR and the incidence of PAEs, as well as examines its correlation with short- to mid-term postoperative mortality.

## 2 Methods

### 2.1 Study population

This retrospective cohort analysis enrolled 363 patients diagnosed with ATAAD complicated by extensive aortic arch pathology who underwent emergency type Ⅱ hybrid aortic arch repair (HAR) at a single center between January 2021 and February 2024. Eligibility criteria for inclusion comprised the following: (1) Age > 18 years; (2) diagnosis of ATAAD confirmed by computed tomography angiography (CTA) with extensive aortic arch pathology; and (3) performance of emergency surgical intervention within 14 days of symptom onset. The exclusion criteria were: (1) Chronic aortic dissection or surgery > 14 days after symptom onset; (2) Death within 24 h after surgery; (3) Iatrogenic or traumatic aortic dissection; (4) Severe organ dysfunction (e.g., hepatic or renal failure); (5) Diseases affecting initial blood cell counts or in-hospital mortality (e.g., malignancies, hematological diseases, infectious diseases); (6) Use of drugs affecting complete blood count (CBC) parameters; (7) Data of incomplete cases. This retrospective study was approved by the Ethics Committee of The First Affiliated Hospital of Soochow University (IRB No. 2025392) with a waiver of informed consent and conducted in accordance with the Declaration of Helsinki (2013 revision).

### 2.2 Data acquisition

The clinical data of the enrolled patients were retrieved from the electronic medical record system, including demographic details (age, sex, etc), admission blood pressure, preoperative comorbid conditions (hypertension, diabetes, coronary heart disease, etc.), time from symptom onset to admission. Imaging findings (aortic valve regurgitation, pericardial and pleural effusions, etc) and preoperative laboratory tests were also recorded. Intraoperative details such as total operative time, cardiopulmonary bypass (CPB) duration, aortic cross-clamp time, and surgical technique were documented. Postoperative data comprised intensive care unit (ICU) stay time, mechanical ventilation time, and the occurrence of postoperative complications. CBC parameters were obtained within 1 h of hospital admission using automated hematology analyzers. Other biochemical markers were measured within 24 h of admission. A follow-up was conducted to document all-cause mortality.

### 2.3 Definition and endpoint

Malperfusion Syndrome (MPS) was identified in patients with reduced perfusion or complete arterial obstruction as evidenced by preoperative CTA, in combination with clinical manifestations such as coma, limb paralysis, or abdominal pain, or supported by elevated cardiac enzyme levels. Preoperative hypotension was defined as a systolic blood pressure <.90 mmHg. Severe AKI was defined as AKI stage III per Kidney Disease: Improving Global Outcomes (KDIGO) criteria (25): serum creatinine (SCr) ≥ 3 times baseline or ≥ 4.0 mg/dL (353.6 μmol/L), or initiation of continuous renal replacement therapy (CRRT) within 1 week postoperatively. Postoperative acute respiratory distress syndrome (ARDS) was diagnosed by a PaO2/FiO2 ratio ≤ 200 mmHg within 24 h after surgery. Mechanical ventilation time was the total duration of tracheal intubation-assisted ventilation.

The primary endpoint of this study was the onset of PAEs during hospitalization, while secondary endpoints included short- and mid-term all-cause deaths. As per the consensus statement issued by the International Aortic Arch Surgery Study Group regarding the grading of complications after aortic arch surgery (26), PAEs were recognized as clinical events corresponding to grade III or higher complications. In this study, PAEs included: (1) CVD complications [malignant arrhythmias, myocardial ischemia, and heart failure needed support with intra-aortic balloon pump (IABP) or extracorporeal membrane oxygenation (ECMO)], (2) respiratory complications (ARDS, reintubation, tracheostomy), (3) new-onset severe AKI, (4) gastrointestinal hemorrhage, (5) wound-related problems needed reoperation for hemostasis or additional surgical therapeutics, (6) death after surgery.

### 2.4 Surgical approach and techniques

Under general anesthesia, emergency one-stage total arch replacement using the type Ⅱ HAR technique was performed through a standard median sternotomy. Femoral arterial cannulation was employed to establish CPB in all patients, with venous drainage via a right atrial two-stage cannula. Our cerebral protection strategy included temperature management, cerebral oximetry, and intraoperative transcranial Doppler (TCD) monitoring. Based on TCD findings, particularly for patients with dissection involving the arch vessels, an additional arterial cannula was selectively placed in the right axillary artery to create a dual-arterial cannulation strategy, ensuring adequate cerebral perfusion. The ascending aorta was replaced by an anastomosing four-branched prosthetic graft to the sinotubular junction. The aortic arch was transected just proximal to the origin of the innominate artery, and the distal end of the prosthesis was sutured in an end-to-end manner to the distal aortic arch. Further sequential anastomosis of the graft branches to the left common carotid artery, left subclavian artery, and innominate artery was performed. Endovascular stent grafting was completed by advancing the endograft retrogradely through the femoral artery access site. The proximal portion of the endograft was anchored to the distal segment of the prosthetic graft, thus completing the arch reconstruction. Surgical management of the aortic root included procedures such as the Bentall procedure, David procedure, Wheat procedure, isolated ascending aortic replacement, or a combination with coronary artery bypass grafting (CABG) and/or other concomitant cardiac valve procedures. Postoperatively, patients were transferred to the ICU for monitoring until clinical stabilization.

### 2.5 Statistical analysis

Statistical analysis was statistically quantified via SPSS v25.0 and R software v4.3.3. Continuous data are presented as mean ± SD (normally distributed) or median (IQR) (non-normally distributed), compared using Student's *t*-test or Mann–Whitney *U*-test, respectively. Categorical variables are summarized as percentages and analyzed by *χ*² test. Patients were stratified into low and high PWR groups based on the median value to ensure balanced group sizes. Receiver operating characteristic (ROC) curve analysis was initially used to examine the discriminative ability of PWR in predicting PAEs among patients with ATAAD. The association between the PWR and PAEs was evaluated using a series of multivariable logistic regression models. A stepwise selection algorithm based on the Akaike Information Criterion (AIC) was employed to optimize model parsimony and performance. To avoid multicollinearity, variables with a variance inflation factor (VIF) ≥ 5 were excluded. The modeling strategy comprised a crude (unadjusted) model, followed by sequentially adjusted models: Model 1 (demographics and comorbidities: age, sex, BMI, hypertension, diabetes); Model 2 (Model 1 + clinical and operative factors: preoperative hypotension, MPS, operative time, CPB duration); and Model 3 (Model 2 + laboratory markers: hemoglobin, AST, SCr, D-dimer). Subgroup analyses (age, sex, BMI, hypertension, time from symptom onset to admission, AST, SCr, D-dimer and CPB duration) assessed effect modification, testing interactions for significance. Kaplan–Meier survival analysis was employed to construct survival curves, with comparative analysis between groups using the log-rank test. The association between PWR and short- and mid-term postoperative mortality was further evaluated using Cox proportional hazards regression analysis. A value of *p* < 0.05 was considered to be statistically significant.

## 3 Results

### 3.1 Basic features of participants

**Figure 1:**
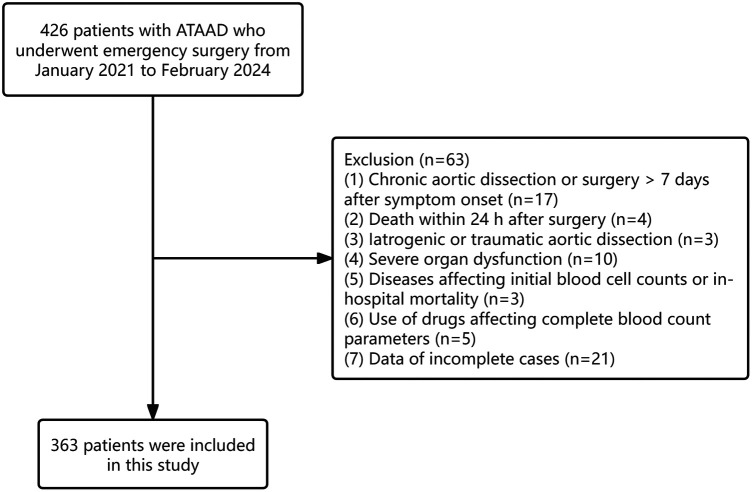
Flow chart of the study.

Approximately 426 patients diagnosed between January 2021 and February 2024 were initially assessed for eligibility. Exclusion criteria were applied to 17 patients with chronic aortic dissection, four individuals died within 24 h postoperatively, three cases involving iatrogenic or traumatic aortic dissection, ten patients presenting with severe organ dysfunction, three patients with conditions affecting baseline hematological parameters or associated with in-hospital mortality, five cases with the use of drugs affecting CBC values, and 21 patients with incomplete clinical records. After these exclusions, 363 patients were ultimately enrolled in the analysis (Figure 1). Of these, 312 were men (85.95%) and 51 were women (14.05%). The median age was 50 years, and hypertension emerged as the most prevalent comorbidity (68.87%).

All patients were assigned to two groups as per the median PWR: the low PWR group (<13.259, *n* = 181) and the high PWR group (≥ 13.259, *n* = 182). As shown in Table 1. Patients in the Low PWR group had higher proportions of preoperative hypotension and MPS, and had a significantly shorter time from symptom onset to admission. The WBC, hemoglobin, ALT, AST, SCr, PT, Fibrinogen, D-dimer, Hs-cTn, and Myoglobin levels were remarkably higher in the low PWR group, while the PLT and CRP level between the two groups was the opposite, and the difference was statistically significant.

**Table 1:** Preoperative and intraoperative clinical characteristics between different PWR groups.

Variables	Total (*n* = 363)	Low PWR (*n* = 181)	High PWR (*n* = 182)	*P* value
Demographics				
Age, y	50 (40, 60)	50 (41, 59)	51 (40, 63)	0.562
Male, *n* (%)	312 (85.95)	162 (89.50)	150 (82.42)	0.052
BMI, kg/m^2^	25.95 (23.79, 29.27)	26.12 (24.15, 28.86)	25.80 (23.47, 29.39)	0.448
Risk factors and comorbidities				
Hypertension, *n* (%)	250 (68.87)	121 (66.85)	129 (70.88)	0.407
Diabetes, *n* (%)	16 (4.41)	8 (4.42)	8 (4.40)	0.991
Coronary heart disease, *n* (%)	15 (4.13)	8 (4.42)	7 (3.85)	0.784
Previous cerebrovascular disease, *n* (%)	8 (2.20)	1 (0.55)	7 (3.85)	0.075
Previous CHF, *n* (%)	18 (4.96)	8 (4.42)	10 (5.49)	0.637
COPD, *n* (%)	8 (2.20)	4 (2.21)	4 (2.20)	1.000
Marfan Syndrome, *n* (%)	15 (4.13)	4 (2.21)	11 (6.04)	0.066
Time from onset to admission, *n* (%)				**0** **.** **003**
<12 h	299 (82.37)	158 (87.29)	141 (77.47)	
12–24 h	29 (7.99)	15 (8.29)	14 (7.69)	
≥ 24 h	35 (9.64)	8 (4.42)	27 (14.84)	
Preoperative hypotension, *n* (%)	24 (6.61)	18 (9.94)	6 (3.30)	**0** **.** **011**
MPS, *n* (%)	77 (21.21)	47 (25.97)	30 (16.48)	**0** **.** **027**
Pericardial effusion, *n* (%)	121 (33.33)	59 (32.60)	62 (34.07)	0.767
Pleural effusion, *n* (%)	62 (17.08)	31 (17.13)	31 (17.03)	0.981
Aortic valve regurgitation (Moderate or above), *n* (%)	44 (12.12)	23 (12.71)	21 (11.54)	0.733
LVEF, %	62.00 (59.00, 65.00)	62.00 (59.00, 65.00)	61.00 (59.00, 64.00)	0.055
Diameter of the ascending aorta, mm	43.00 (40.00, 48.00)	44.00 (40.00, 48.00)	43.00 (39.00, 49.75)	0.776
Diameter of the aorta sinus, mm	41.00 (38.00, 44.00)	41.00 (38.00, 45.00)	40.00 (37.25, 44.00)	0.211
WBC, ×10^9^/L	13.10 (10.69, 16.23)	15.38 (12.46, 18.36)	11.49 (8.91, 13.92)	**<** **.** **001**
Hemoglobin, g/L	135.74 ± 16.32	137.87 ± 15.05	133.62 ± 17.27	**0** **.** **013**
PLT, ×10^9^/L	183.02 ± 60.56	156.33 ± 44.59	209.56 ± 62.75	**<** **.** **001**
CRP, mg/L	4.00 (1.77, 14.18)	3.16 (1.48, 8.42)	5.34 (1.95, 19.19)	**0** **.** **003**
ALT, U/L	22.20 (14.80, 40.00)	24.30 (16.00, 45.80)	21.25 (13.83, 35.58)	**0** **.** **009**
AST, U/L	21.40 (15.80, 31.10)	23.50 (17.40, 39.10)	19.40 (14.72, 26.00)	**<** **.** **001**
Albumin, g/L	38.98 ± 4.40	39.39 ± 4.31	38.57 ± 4.47	0.076
SCr, μmol/L	82.40 (65.25, 110.05)	91.40 (68.00, 117.20)	77.15 (62.05, 103.50)	**<** **.** **001**
CysC, mg/L	0.98 (0.85, 1.21)	1.01 (0.87, 1.29)	0.97 (0.84, 1.18)	0.070
PT, sec	14.20 (13.60, 14.80)	14.30 (13.80, 15.20)	14.00 (13.40, 14.50)	**<** **.** **001**
APTT, sec	35.50 (32.20, 39.25)	35.70 (32.20, 38.60)	35.40 (32.35, 39.60)	0.752
Fibrinogen, g/L	2.57 (1.92, 3.23)	2.21 (1.63, 2.72)	2.91 (2.36, 3.72)	**<** **.** **001**
D-dimer, ug/mL	9.38 (2.62, 20.00)	16.52 (5.08, 20.00)	4.62 (1.79, 14.39)	**<** **.** **001**
hs-cTnT, pg/mL	13.12 (8.72, 30.65)	16.82 (9.63, 41.15)	12.20 (8.06, 21.57)	**<** **.** **001**
Myoglobin, ng/mL	48.91 (29.45, 88.76)	63.01 (34.87, 119.00)	37.98 (24.40, 63.87)	**<** **.** **001**
NT-proBNP, pg/mL	147.80 (65.24, 383.50)	160.90 (72.80, 396.00)	133.25 (56.11, 357.25)	0.176
Intraoperative data				
Bentall, *n* (%)	50 (13.77)	26 (14.36)	24 (13.19)	0.745
David, *n* (%)	5 (1.38)	3 (1.66)	2 (1.10)	0.995
Wheats, *n* (%)	13 (3.58)	4 (2.21)	9 (4.95)	0.161
CABG, *n* (%)	13 (3.58)	9 (4.97)	4 (2.20)	0.155
Mitral surgery, *n* (%)	1 (0.28)	1 (0.55)	0 (0.00)	0.499
Operative time, h	7.58 (6.33, 8.71)	7.67 (6.50, 8.83)	7.46 (6.00, 8.57)	0.097
CPB duration, min	180.00 (151.50, 218.00)	185.00 (154.00, 214.00)	180.00 (148.50, 220.00)	0.341
Aortic crossclamp time, min	106.00 (83.00, 135.00)	106.00 (87.00, 134.00)	107.50 (80.00, 137.50)	0.533

PWR, platelet-to-white blood cell ratio; BMI, body mass index; CHF, chronic heart failure; COPD, chronic obstructive pulmonary disease; MPS, malperfusion syndrome; LVEF, left ventricular ejection fractions; WBC, white blood cell; PLT, platelet; CRP, C-reactive protein; ALT, alanine aminotransferase; AST, aspartate aminotransferase; SCr, serum creatinine; CysC, cystatin C; PT, prothrombin time; APTT, activated partial thromboplastin time; hs-cTnT, high-sensitivity cardiac troponin T; NT-proBNP, N-terminal pro-brain natriuretic peptide; CPB, cardiopulmonary bypass; CABG, coronary artery bypass grafting. Bold values indicate a *p*-value less than 0.05, indicating a statistically significant difference between groups.

### 3.2 PAEs between different PWR groups

As shown in Table 2, Patients with ATAAD in the low PWR group had significantly higher rates of PAEs than the high PWR group (41.99% vs. 21.43%; *p* < 0.001), particularly ARDS, severe AKI, CRRT requirement, and spinal cord injury. Moreover, the duration of mechanical ventilation was significantly prolonged in the low PWR cohort (median 44.40 vs. 37.20 h, *p* < 0.001). No statistically significant variations were found between both groups in the case of the incidence of postoperative myocardial ischemia, ventricular arrhythmia, use of IABP or ECMO, secondary endotracheal intubation, tracheostomy, cerebrovascular accident, gastrointestinal hemorrhage, septic shock, or extended ICU stay (all *p* ≥ 0.05).

**Table 2:** PAEs between different PWR groups.

Variables	Total (*n* = 363)	Low PWR (*n* = 181)	High PWR (*n* = 182)	*P* value
PAEs, *n* (%)	115 (31.68)	76 (41.99)	39 (21.43)	**<**.**001**
Myocardial ischemia, *n* (%)	10 (2.75)	7 (3.87)	3 (1.65)	0.332
Ventricular arrhythmia, *n* (%)	7 (1.93)	5 (2.76)	2 (1.10)	0.441
IABP/ECMO, *n* (%)	3 (0.83)	3 (1.66)	0 (0.00)	0.244
ARDS, *n* (%)	79 (21.76)	54 (29.83)	25 (13.74)	**<**.**001**
Secondary intubation, *n* (%)	14 (3.86)	8 (4.42)	6 (3.30)	0.578
Tracheotomy, *n* (%)	31 (8.54)	20 (11.05)	11 (6.04)	0.088
Severe AKI, *n* (%)	49 (13.50)	37 (20.44)	12 (6.59)	**<**.**001**
CRRT, *n* (%)	40 (11.02)	31 (17.13)	9 (4.95)	**<**.**001**
Stroke, *n* (%)	25 (6.89)	16 (8.84)	9 (4.95)	0.143
Spinal cord injury, *n* (%)	9 (2.48)	10 (5.52)	3 (1.65)	**0**.**047**
Gastrointestinal bleeding, *n* (%)	13 (3.58)	7 (3.87)	2 (1.10)	0.174
Septic shock, *n* (%)	27 (7.44)	17 (9.39)	10 (5.49)	0.157
Secondary thoracotomy, *n* (%)	7 (1.93)	2 (1.10)	5 (2.76)	0.445
Mechanical ventilation time, h	39.00 (22.20, 66.00)	44.40 (28.20, 73.20)	37.20 (20.55, 59.70)	**0**.**011**
ICU stay time, d	3.90 (2.70, 6.00)	4.20 (3.00, 6.60)	3.60 (2.70, 5.33)	0.050
In-hospital Mortality, *n* (%)	31 (8.54)	25 (13.81)	6 (3.30)	**<**.**001**

PAEs, postoperative adverse events; PWR, platelet-to-white blood cell ratio; IABP, intra-aortic balloon pump; ECMO, extracorporeal membrane oxygenation; ARDS, acute respiratory distress syndrome; AKI, acute kidney injury; CRRT, continuous renal replacement therapy; ICU, intensive care unit.

Bold values indicate a *p*-value less than 0.05, indicating a statistically significant difference between groups.

### 3.3 Association between the PWR and PAEs

Univariate logistic regression analysis identified preoperative PWR as an effective predictor of PAEs in patients with ATAAD (OR: 2.65, 95%CI: 1.67–4.21, *p* < 0.001). To further explore this association, multivariate logistic regression models were adjusted for potential confounding variables (Table 3). After this adjustment, low preoperative PWR remained independently associated with an increased risk of PAEs. In Model 1, the OR was 2.98 (95% CI: 1.83–4.84, *p* < 0.001); in Model 2, the OR was 2.55 (95% CI: 1.51–4.30, *p* < 0.001); and in Model 3, the OR was 2.20 (95% CI: 1.24–3.91, *p* = 0.007).

**Table 3:** Multivariable logistic analysis of the association between PWR and PAEs in patients with ATAAD.

PAEs	High PWR	Low PWR	*P* value
		OR (95%CI)	
Crude model	Ref	2.65 (1.67–4.21)	**<**.**001**
Model 1	Ref	2.98 (1.83–4.84)	**<**.**001**
Model 2	Ref	2.55 (1.51–4.30)	**<**.**001**
Model 3	Ref	2.20 (1.24–3.91)	**0**.**007**

OR, odds ratio; CI, confidence interval; PWR, platelet-to-white blood cell ratio; PAEs, postoperative adverse events; ATAAD, acute type A aortic dissection; Model 1 was adjusted for age, sex, BMI, hypertension, diabetes; Model 2 was adjusted for Model 1 and preoperative hypotension, MPS, operative time, CPB duration; Model 3 was adjusted for Model 2 and hemoglobin, AST, SCr, D-dimer.

Bold values indicate a *p*-value less than 0.05, indicating a statistically significant difference.

**Figure 2:**
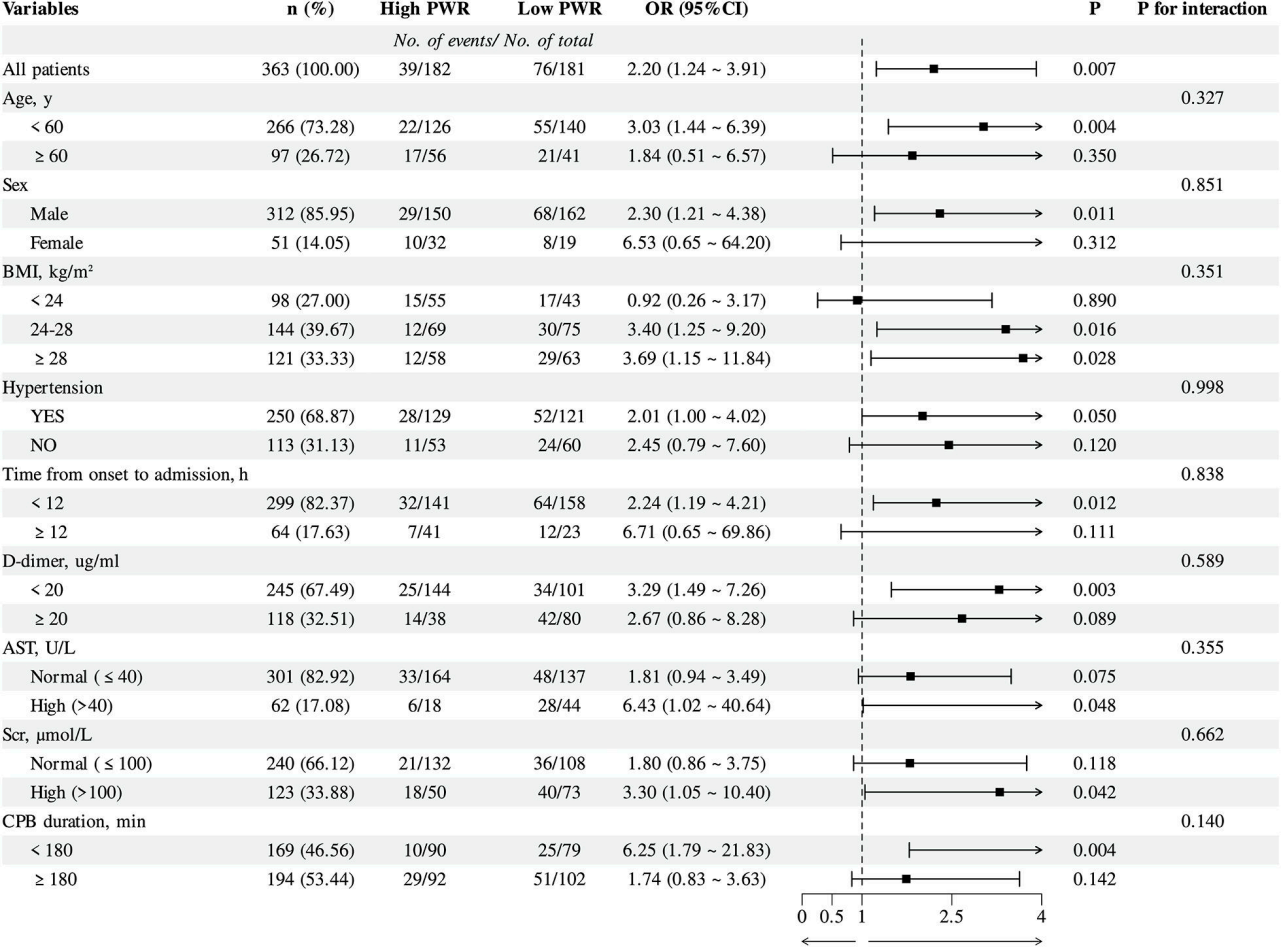
Subgroup analysis of logistic regression model 3 for PAEs with the PWR in patients with ATAAD. PWR, Platelet-to-White Blood Cell Ratio; PAEs, postoperative adverse events; ATAAD, acute type A aortic dissection; Models adjusted for the same covariates as in Model 3 (Table 3), except for the stratification variable.

To explore whether the link between PWR and the risk of PAEs varied across clinical subgroups, stratified analyses were carried out by age, sex, BMI, hypertension status, time from symptom onset to admission, AST, SCr, D-dimer levels, and CPB duration (Figure 2), and no statistically significant interactions were identified among the subgroups (all *p* > 0.05).

**Figure 3:**
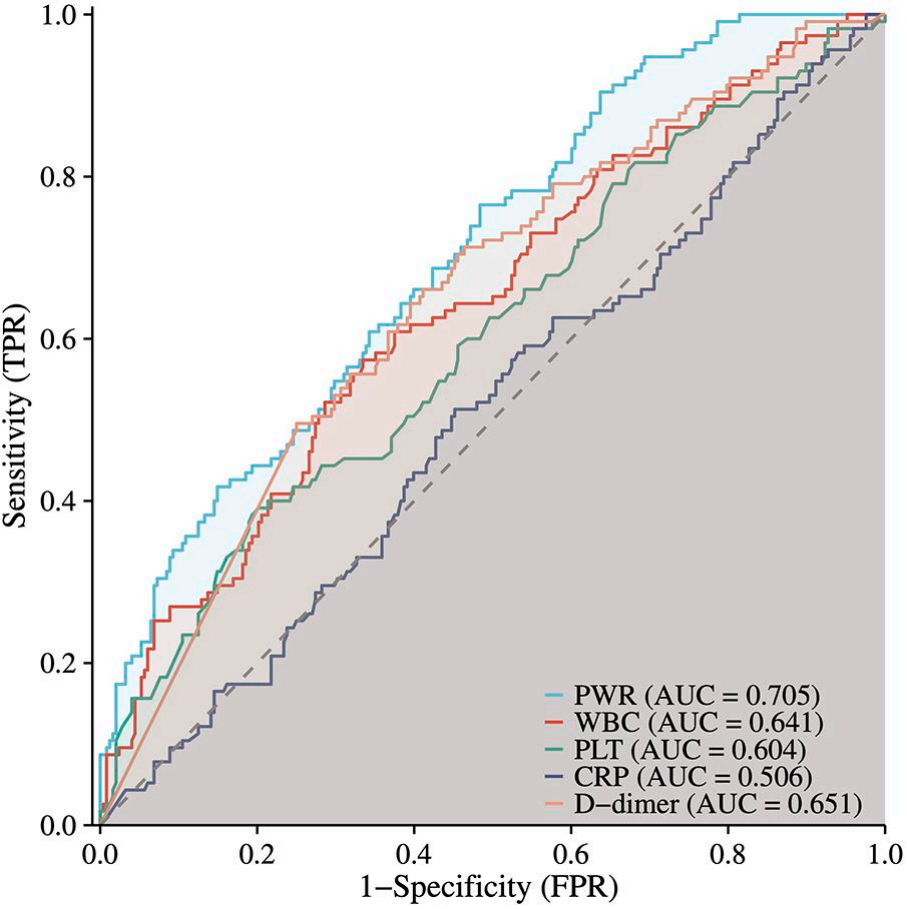
ROC curve of PWR and other clinical indicatorsin predicting PAEs. ROC, receiver operating characteristic; PWR, platelet-to-white blood cell ratio.

To evaluate the prognostic utility of the PWR, ROC curve analysis was compared with commonly used clinical markers of inflammatory activity (WBC, CRP) and coagulation status (PLT, D-dimer). Among these indicators, PWR demonstrated higher predictive performance for PAEs. The areas under the curve (AUC) for PWR was 0.705 (95% CI: 0.649–0.760), suggesting moderate discriminative ability (Figure 3).

### 3.4 Association between PWR and short- and mid-term mortality

The 1-year follow-up achieved a complete survival data acquisition rate of 100%. Patients classified in the low PWR group demonstrated markedly elevated rates of in-hospital mortality (13.81% vs. 3.30%, *p* = 0.001), 90-day mortality (14.36% vs. 4.40%, *p* < 0.001), and all-cause mortality at one year (16.38% vs. 5.56%, *p* = 0.001) in comparison to individuals in the higher PWR group.

**Figure 4:**
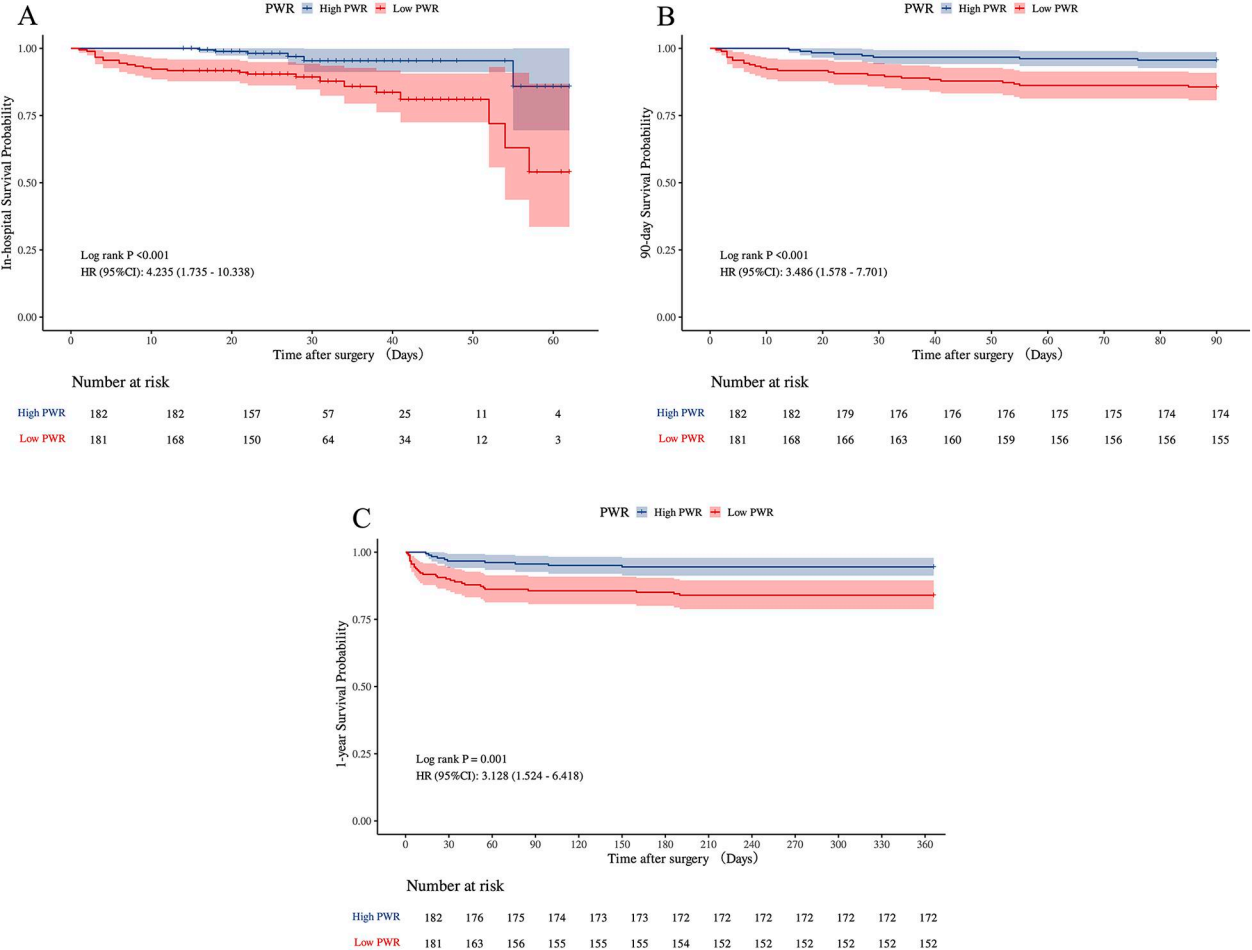
Kaplan–Meier survival analyses for in-hospital, 90-day, and 1-year mortalities of ATAAD patients in PWR group **(A–C)**. ATAAD, acute type A aortic dissection; PWR, platelet-to-white blood cell ratio.

Similarly, K-M survival analyses revealed significant differences in overall survival probabilities between the low and high PWR groups across all evaluated endpoints. As depicted in Figures 4A–C, the low PWR group showed considerably higher risks of hospitalized (*p* < 0.001), 90-day (*p* < 0.001), and 1-year (*p* = 0.001) deaths.

To further examine the link between PWR and early to mid-term mortality, Cox proportional hazards regression models were applied, as summarized in Table 4. In comparison to the high PWR group, patients in the low PWR group showed significantly elevated hazard ratios (HR) across all mortality endpoints. For in-hospital mortality, the crude model reported an HR of 4.24 (95% CI: 1.74–10.34, *p* = 0.002), whereas the fully adjusted model indicated an HR of 4.02 (95% CI: 1.54–10.45, *p* = 0.004). Analogous associations were detected for 90-day and 1-year mortality outcomes, thus highlighting the prognostic relevance of low preoperative PWR as a distinct predictor of short- and mid-term mortality in ATAAD patients.

**Table 4:** Multivariable Cox regression analysis of the association between PWR and short- and mid-term mortality in patients with ATAAD.

Category	Models	High PWR	Low PWR	*P* value
			HR (95%CI)	
In-hospital mortality	Crude model	Ref	4.24 (1.74–10.34)	**0**.**002**
	Model 1	Ref	4.59 (1.87–11.30)	**<**.**001**
	Model 2	Ref	3.77 (1.49–9.49)	**0**.**005**
	Model 3	Ref	4.02 (1.54–10.45)	**0**.**004**
90-day mortality	Crude model	Ref	3.49 (1.58–7.70)	**0**.**002**
	Model 1	Ref	3.79 (1.71–8.41)	**0**.**001**
	Model 2	Ref	3.07 (1.34–7.04)	**0**.**008**
	Model 3	Ref	3.43 (1.43–8.23)	**0**.**006**
1-year mortality	Crude model	Ref	3.13 (1.52–6.42)	**0**.**002**
	Model 1	Ref	3.38 (1.64–6.98)	**<**.**001**
	Model 2	Ref	2.86 (1.35–6.06)	**0**.**006**
	Model 3	Ref	3.07 (1.39–6.78)	**0**.**006**

HR, hazard ratio; CI, confidence interval; PWR, platelet-to-white blood cell ratio; ATAAD, acute type A aortic dissection; Model 1was adjusted for age, sex, BMI, hypertension, diabetes; Model 2 was adjusted for Model 1 and preoperative hypotension, MPS, operative time, CPB duration; Model 3 was adjusted for Model 2 and hemoglobin, AST, SCr, D-dimer.

Bold values indicate a *p*-value less than 0.05, indicating a statistically significant difference.

## 4 Discussion

ATAAD is recognized as a critical CVD emergency associated with significant mortality. Thus, timely detection of high-risk patients is essential for optimizing clinical outcomes. The present retrospective study evaluated clinical data from 363 ATAAD patients who underwent HAR surgery. It was the first time that demonstrated a remarkable correlation between preoperative PWR and the incidence of PAEs in this patient population. The findings indicated that individuals presenting with lower PWR values (< 13.259) at admission experienced a higher frequency of PAEs during the perioperative period. Importantly, even after adjustment for potential confounders, reduced PWR remained a distinct predictor of PAE risk. Moreover, short-and mid-term survival analyses revealed markedly reduced survival rates in the low PWR group relative to those with higher PWR values, as evidenced by significantly elevated in-hospital, 90-day, and 1-year all-cause mortality rates. These observations underscore the clinical utility of preoperative PWR in identifying high surgical-risk patients with ATAAD.

The PWR has shown key prognostic value in CVD and inflammatory complications, including cardiac arrest, acute decompensated heart failure, ST-segment elevation myocardial infarction, stroke, and COVID-19 (21, 22, 27–29). A multicenter cohort study revealed that PWR showed a stronger correlation with patient mortality than other blood cell count markers and their ratios in four major acute inflammatory diseases (acute heart failure, myocardial infarction, COVID-19, and stroke) (30). Moreover, PWR is also associated with postoperative problems and mortality. Analysis of the MOVER database surgical cohort (31), spanning procedures from minor skin excisions to major operations such as mitral valve replacements, demonstrated that PWR outperformed traditional blood count metrics and ratios in predicting mortality (30). Previous evidence has shown that preoperative PWR serves as a distinct prognostic parameter for postoperative AKI in individuals undergoing cerebral aneurysm surgery, with reduced PWR levels substantially associated with an elevated risk of postoperative AKI (32). In the ATAAD, PWR has also been shown to possess prognostic relevance for perioperative mortality after surgical intervention. The findings from a multicenter cohort study conducted in China reported an effective correlation between PWR and 30-day postoperative mortality in ATAAD patients, with higher predictive performance than conventional individual hematological indices (23, 24). This study found that patients with low PWR had a significantly higher PAEs incidence, particularly in complications such as postoperative ARDS, severe AKI, CRRT requirement, and spinal cord injury. They also required longer mechanical ventilation times, indicating a more complex and challenging postoperative recovery process. ROC curve analysis indicated superior discriminatory power for PWR compared to individual biomarkers (WBCs, PLT, CRP, and D-dimer). This finding underscores the enhanced utility of PWR in preoperative risk stratification for ATAAD patients, consistent with prior evidence.

Furthermore, a longitudinal cohort study evaluating systemic inflammatory and coagulation trajectories in ATAAD patients undergoing total arch replacement with FET implantation reported that a declining PWR pattern, characterized by either a progressive increase in WBC or a gradual reduction in PLT count, was significantly associated with elevated short- and mid-term mortality (33). This study included ATAAD patients who underwent HAR surgery. Kaplan–Meier survival analysis demonstrated consistently lower survival probabilities across all time points in the low PWR group. Cox proportional hazards model revealed that after adjustment for possible confounding variables, a reduced preoperative PWR persisted as an independent predictor of both short- and mid-term mortality. These results are consistent with previous research, further supporting the prognostic utility of PWR in predicting adverse postoperative outcomes in patients with ATAAD undergoing HAR procedures.

PWR represents an integrated index comprising two main physiological pathways: the inflammatory immune response and the coagulation cascade. WBCs are primary mediators of systemic inflammation. PLTs, primarily recognized for their role in hemostasis, also actively contribute to inflammatory signaling through the release of cytokines and direct interactions with immune effector cells. The pathogenesis and clinical progression of ATAAD are closely related to dysregulation within these two systems. Thus, PWR may be an effective marker of the complex interaction between inflammation and coagulation (34). The aortic tissue damage caused by dissection and the thrombus in the false lumen may induce an inflammatory response, during which WBCs, once activated, release various inflammatory mediators and proteases. These inflammatory mediators (IL-6 and TNF-α), along with proteases like matrix metalloproteinases (MMPs), can disrupt the structural integrity of the aortic wall, thus increasing the risk of aortic dissection progression (35, 36). WBCs (neutrophils and macrophages) have been detected in the torn aortic tissues. Compared to asymptomatic and clinically stable patients, those with marked clinical manifestations and disease progression demonstrate increased inflammatory cell activity within the aortic wall (37). Previous studies have identified that the WBC count obtained within 24 h of admission in cases of ATAAD represents a potent and distinct prognostic indicator of hospitalized mortality and composite adverse outcomes (38). Additionally, preoperative leukocytosis has been identified as an independent predictor of postoperative hypoxemia in this population (39), and as a significant risk factor for tracheostomy requirement (40). PLTs also play a pivotal role in the pathophysiology of ATAAD. Inflammatory processes contributing to thrombosis within the false lumen can activate PLTs, leading to their consumption and resulting in reduced circulating PLT counts. This consumptive process has been associated with adverse clinical outcomes (41–43). Further, activated PLTs release various inflammatory mediators into the local microenvironment, thus highlighting systemic inflammation and contributing to organ dysfunction (44). Therefore, low PLT levels may be an effective marker for inflammatory and thrombotic activity in aortic dissection (43, 45). Low preoperative PLT counts have been independently associated with increased in-hospital mortality and reduced mid-term survival in ATAAD patients undergoing surgical repair (15). Furthermore, reduced PLT levels at admission have been correlated with a higher incidence of postoperative pulmonary diseases, such as pneumonia, which may in turn prolong the duration of ICU stay (46).

The association between low PWR and the onset of PAEs and negative outcomes in ATAAD patients postoperatively likely reflects the dual dysregulation of coagulation and inflammation in the pathogenesis of ATAAD. Low PWR may indicate platelet consumption or an elevated white blood cell count. This aligns with previous evidence on the coagulation-inflammation interaction driving vascular injury in ATAAD (10). These factors collectively contribute to an elevated vulnerability in patients with ATAAD to various complications, including impaired organ perfusion and extensive inflammatory injury, ultimately resulting in increased mortality. Surgical procedures involving aortic manipulation and the use of cardiopulmonary bypass are known to trigger systemic inflammatory response syndrome (SIRS), which can further compromise physiological stability. In patients with higher baseline inflammatory sensitivity (as reflected by low PWR), the risk of developing severe postoperative complications (ARDS and AKI) is substantially elevated. These complications are frequently associated with prolonged mechanical ventilation, extended ICU stays, and worsened overall survival outcomes (47).

### 4.1 Advantages

Firstly, the calculation of the PWR relies solely on parameters derived from standard complete blood count tests, eliminating the need for specialized equipment and enhancing applicability in resource-limited settings. Secondly, this study found that PWR is primarily associated with PAEs, such as ARDS, severe AKI, CRRT requirement, and spinal cord injury, further analysis can be conducted to explore the predictive role of PWR in these complications. Particularly, the subgroup analysis confirmed that reduced PWR consistently predicted the incidence of PAEs across all predefined strata (age, sex, BMI, time from symptom onset to admission, hepatic and renal function, CPB duration; *p* > 0.05 for interaction). This indicates that the prognostic value of low PWR reflects an inherent pathophysiological state, independent of surgical complexity or baseline organ function.

### 4.2 Limitations and prospects

Despite providing compelling evidence for the association between the PWR and surgical outcomes in ATAAD patients, this study has limitations. First, its retrospective, single-center design risks selection bias and limits generalizability, while residual confounding may persist despite adjusted models. Second, the modest sample size reduced power to detect associations with rare complications or conduct thorough subgroup analyses. Third, the absence of longitudinal PWR data precludes assessment of dynamic fluctuations and their prognostic relevance. Fourth, due to the relatively short follow-up period and the inherent challenges in retrospectively capturing complete rehospitalization information, we were unable to perform a competing risk analysis using the Cumulative Incidence Function (CIF) to evaluate rehospitalization with death as a competing risk. Future multicenter prospective studies with larger cohorts and longer follow-up are needed to validate these findings. Comprehensive research should also focus on exploring the role of PWR in clinical staging (e.g., preoperative diagnosis) and its underlying molecular mechanisms, while incorporating systematic rehospitalization data and advanced methods like CIF to definitively establish its prognostic utility for long-term outcomes.

## 5 Conclusion

This study concluded that the PWR is a simple, readily obtainable, and clinically informative preoperative biomarker in ATAAD patients undergoing surgery. Its reduced level was independently related to an increased risk of PAEs and elevated short- to mid-term mortality. Thus, the identification of patients with low PWR may allow for the timely implementation of targeted interventions to improve postoperative outcomes and overall prognosis in this high-risk population.

## Data Availability

The original contributions presented in the study are included in the article/Supplementary Material, further inquiries can be directed to the corresponding author.
